# 20 Years of the International Workshop on Osteoarthritis imaging (IWOAI): From a Small Pre-Alpine Village to a Global Community

**DOI:** 10.1016/j.ostima.2026.100396

**Published:** 2026-05-20

**Authors:** Felix Eckstein, David J. Hunter, Ali Guermazi

**Affiliations:** a Research Group for Musculoskeletal Imaging, Center for Anatomy and Cell Biology, Paracelsus Medical University, Salzburg, Austria; b Ludwig Boltzmann Institute for Arthritis and Rehabilitation; c Chondrometrics GmbH, Freilassing, Germany; d Sydney Musculoskeletal Health, Kolling Institute, Faculty of Medicine and Health, The University of Sydney, Sydney, NSW, Australia; e Rheumatology Department, Royal North Shore Hospital, St Leonards, NSW 2065 Australia; f Department of Radiology, VA Boston Healthcare System, Boston University School of Medicine, Boston, MA, USA; g Boston Imaging Core Lab (BICL), Boston, MA, USA

**Keywords:** Imaging, Workshop, Osteoarthritis, Structure modification, Community, Anniversary

## Abstract

**Introduction:**

The International Workshop on Osteoarthritis Imaging (IWOAI) was founded in 2007 to create a focused scientific forum dedicated to imaging (biomarkers) in osteoarthritis. In contrast to large international congresses, the workshop was conceived as a small and interactive meeting encouraging open discussion and interdisciplinary exchange among researchers from academia and industry.

**Perspective:**

Since its first meeting in 2007 in the remote pre-Alpine village of Ainring, IWOAI has evolved into an annual global series of workshops bringing together international experts from many different disciplines. Despite its worldwide expansion, the workshop has remained limited in size, with up to 150 participants (South Carolina in 2012) but often smaller. The retreat-like format has allowed ample time for discussion and personal interaction. It has fostered a collaborative environment in which methodological challenges, emerging imaging technologies, publicly accessible cohorts and image repositories (e.g. the Osteoarthritis Initiative), and regulatory aspects of imaging biomarkers are discussed in depth. Informal interactions during social events and post-conference excursions have strengthened professional networks, collaborations, and personal friendships across disciplines and institutions. Over time, the workshop community also contributed to strengthening the field institutionally by establishing the International Society for Osteoarthritis Imaging (ISOAI) in 2019.

**Outlook:**

As imaging technologies, AI-based automated image analysis, and data science continue to advance, imaging biomarkers are expected to play an increasingly important role in osteoarthritis clinical research and therapeutic development. Continued collaboration among academic researchers, clinicians, industry scientists and regulators will remain essential for translating imaging advances into meaningful clinical applications, trials, and ultimately disease-modifying therapy.

## Introduction

Scientific progress is often associated with large international congresses and consortia. Yet some of the most striking advances in emerging fields originate from smaller gatherings with ample time to discuss ideas in depth with an open mind in a collegial and friendly atmosphere. Meetings always meant to be “meet”-ings, not “speak”-ings, and whilst organizers often seem to think the scientific program is the secret to success, its true strength lies in its attendees. The International Workshop on Osteoarthritis Imaging (IWOAI) was founded with precisely this philosophy. Since its birth in the small pre-Alpine village of Ainring (Germany) in 2007 (Table 1), it brought together researchers across disciplines, to discuss development and application of imaging biomarkers in osteoarthritis (OA).

**Table 1:** Locations, Organizers, Themes and Special Personal Memories of IWOAI Events 2007-2026.

Year	Location	Main Organizer(s)	Theme / Focus	Most special memories
2007	Ainring Germany	Felix Eckstein	Imaging-based measures of OA	20° temp. rise during the workshop and rescue alpine hike to Untersberg mountain
2008	Beverly USA	David Hunter Deb Burstein	Re-examining para-digms in OA imaging	Amphibious vehicles that can master the green and the red universe
2009	York UK	Philip Conaghan	Capitalising on the knowledge investment	National Railway Museum and a planetarium where someone fell asleep (and everyone knew)
2010	Vancouver Canada	David Wilson	Joint mechanics and OA imaging	Spectacular campus and fantastic whale watching
2011	Salzburg Austria	Felix Eckstein	From joint anatomy to clinical outcomes	PMU courtyard concert (Claudia Korek) and mountain tour with zero visibility
2012	Hilton Head, USA	David Hunter	Imaging biomarker validation	Beach and largest meeting ever (150?) (joint with molecular marker group)
2013	Djerba, Tunisia	Ali Guermazi	Multimodal imaging in OA	Visiting Darth Vader in the desert, and trying to convince Yoda to consult on the green/red Universe question.
2014	Reykjavik, Iceland	Ali Guermazi	Moving OA imaging forward	Splendid Blue Lagoon, GER:BRA 7:1 in the WC, and many pretty waterfalls
2015	Pacific Grove, USA	Garry Gold Michael Nevitt	Advances in MRI biomarkers	17-Mile Drive scenic road through Pebble Beach and Pacific Grove
2016	Oulu Finland	Miika Nieminen, Martin Englund	Imaging pheno-types & modeling	Midnight sun & beer on market place, air guitar and sauna during dinner
2017	Sydney Australia	David Hunter, James Linklater	Bridging clinical needs and technology	Vivid Sydney festival and never-ending private invitations by David
2018	Menton France	Ali Guermazi	Structural imaging biomarkers	IWOAI at the sea, Mediterranean dinners; France winning the (soccer) world cup
2019	Charlottetown, Canada	J.D. Johnston, Emily McWalter	Linking structural and clinical imaging	Harbour boat tour and brilliant post-conference bike trip
2020	Salzburg Austria	Felix Eckstein	Multitissue imaging in osteoarthritis	The small (but hybrid) „Covid“-Meeting in liberal Austria
2021	Rotterdam Netherlands	Edwin Oei, Jos Runhaar	Multifaceted OA imaging	Great venue with splendid harbour view and gala dinner on the SS Rotterdam
2022	Tokyo Japan	Nobutake Ozeki, Ichiro Sekiya	Imaging and treat–ment integration	Historical Tokyo boat tour on the Sumida river with amazing food
2023	Lausanne Switzerland	Patrick Omoumi Julien Favre	Imaging of a multi-faceted disease	A city like a treadmill, Olympic Museum, and Montreux Jazz Festival
2024	Marrakech, Morocco	Ali Guermazi	Structure and pain in OA	Medina cultural tour, spectacular markets, and Essaouira excursion
2025	Cambridge UK	Tom Turmezei Cecilia Bassett	Anatomy, structure and function in OA	Typical English weather (30°), college dinner, pubs, and homemade wine
2026	Munich Germany	Frank Roemer, Wolfgang Wirth	Tissues: linking them all together	Endless beer between Hofbräuhaus and Kloster Andechs (monastery)

There were a couple more unique organizing points from the start: IWOAI was more about imaging science and its application to large cohorts and was less about the technical details of image acquisition, although the latter was always welcome. It focused on the role of imaging as biomarkers to advance OA research and support clinical trials. Participants from industry were therefore particularly welcome, without drawing artificial academic borders. As much of the applied imaging research takes place in the context of clinical trials, the meeting enjoyed an open and constructive relationship with scientists from industry (or participants with a stake in both worlds), without making them feel their funding was more important than their questions. Last, the workshop has a strong history of granting poster and young investigator awards, and of creating an atmosphere where the experts are more approachable for early-career scientists than at the larger and more formal meetings.

Already during the first evening of the first IWOAI at the remote “Reiteralm” (above the roofs of Salzburg – Fig. 1), discussions about segmentation, imaging biomarkers, methodological challenges and regulatory aspects of clinical trials readily extended beyond the afternoon session to the dinner table. That evening, it became crystal clear that the format itself created an unusually productive climate for scientific openness, exchange, and progress. Most importantly, participants left not only with new knowledge, but also with lasting professional relationships and friendships.

**Fig. 1:**
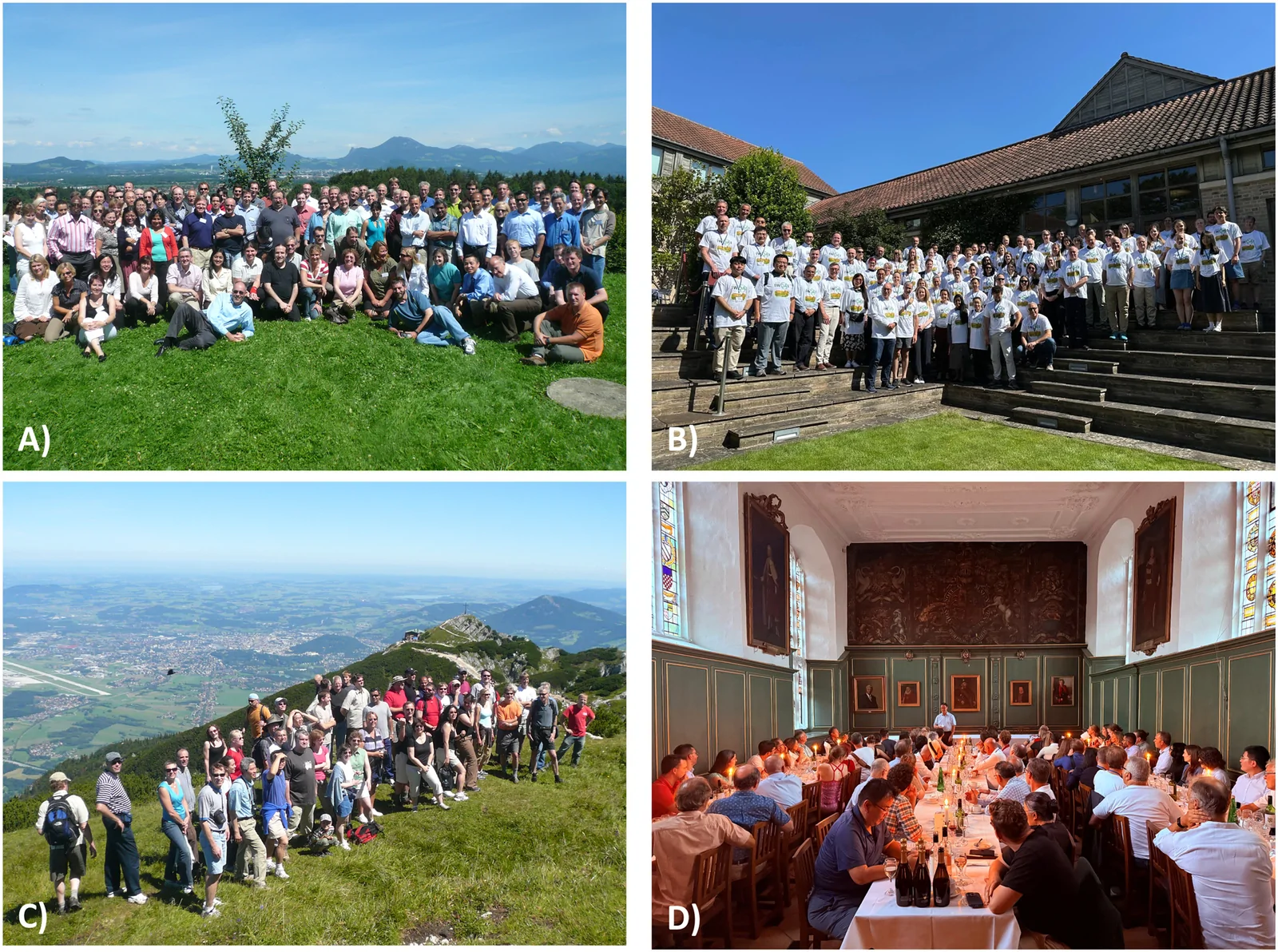
Group photos of the Workshops in 2007 (outside the conference venue at Reiteralm in Ainring, Germany) (a) and 2025 (in the courtyard of Cripps Court, Magdalene College, Cambridge UK) (b), post conference tour in 2007 (at the top of Untersberg mountain near Salzburg, Austria) (c) and conference dinner in 2025 (Benson Hall, Magdalene College, Cambridge, UK) (d).

Nearly two decades later, what began as a small meeting in the Bavarian countryside has developed into an annual global series of workshops, attracting participants from across the world, while maintaining its very spirit and character.

## Origins and first IWOAI

Several developments in the early 2000s motivated the creation of the workshop. At that time, imaging was becoming increasingly central to OA research. Structural imaging was evolving from a primarily diagnostic tool into a powerful instrument for semiquantitative and quantitative assessment. Structural evaluation of multiple joint tissues, e.g. cartilage morphometry and compositional MRI, began to provide new insights into disease mechanisms and progression, and created optimism that imaging biomarkers could enable smaller, faster, and more effective clinical trials of disease modification as hitherto possible, targeting structure as well as symptoms, i.e. the illness associated with OA as a disease.

At the same time, the field became more interdisciplinary. Advances in imaging required close collaboration between radiologists, physicists, imaging scientists, biomedical engineers, biomechanists, clinicians, epidemiologists, statisticians, clinical development scientists, clinical trialists, regulatory specialists, and other experts. The IWOAI originators acknowledged that effective imaging biomarker research cannot take place without implementation in large interventional trials, most often originated, designed, carried out, and published by scientists working in industry. Yet the various stakeholders in this process rarely met, and when they did, interactions were usual brief, since the large conferences leave little time for interactions. Thus, a growing sense developed, that a smaller, focused workshop could provide an important and more enjoyable forum for exchange.

The first IWOAI workshop was planned to take place at Paracelsus Medical University (PMU) in Salzburg, but an unexpected renovation of the library required finding a new venue. What was originally perceived as a disaster by the local team turned out to be a fortunate coincidence. Participants spent several days in a secluded place in a beautiful, quiet Alpine setting, away from the distractions of large venues and conference cities (Fig. 1). The success of this format became immediately apparent. Discussions were lively and interdisciplinary, continuing long after the official sessions and extending into the post-conference tour on Mt Untersberg (Fig. 1). Many participants later described the meeting as unusually productive and intellectually stimulating. Many have returned year after year because of the workshop’s unique scientific and social atmosphere.

## A Meeting Carried Forward by Its Organizers – The Global Journey

One of the most remarkable aspects of IWOAI has been the way in which it has been sustained by a succession of dedicated organizers (Table 1). Each year, one or two colleagues in the field have hosted the meeting and shaped the scientific program together with a scientific committee. While each workshop has developed its own character, organizers have consistently had the freedom to select a main topic, the invited speakers, and define the themes of the pre-congress workshops. At the same time they have consistently preserved the core principles that define the meeting. Further, all hosts incorporated distinctive social and cultural elements, reflecting the local environment. These experiences have become an integral part and have developed into a particular strength of the workshop. At the first round birthday meeting (No 10) in Oulu, Finland, for instance, the sun never set over beer drinking on the central Market Place, and the Finnish Sauna and swim in the adjacent river during the conference dinner was a real local highlight (Table 1).

A characteristic tradition of IWOAI has been the full-day post-conference excursions. These have ranged from alpine hikes to desert tours and cultural visits, or explorations of historic cities, and provide a great opportunity for informal discussion after the participants have had time to reflect on the scientific presentations. These day trips create relaxed opportunities for interaction between junior and senior researchers (Fig. 1). For the founder of the meeting and the first organizational successors, it has been particularly rewarding to see how colleagues around the world have embraced this concept and carried the workshop forward with enthusiasm and creativity.

Since its inception in 2007, the workshop has travelled widely across Europe, North America, Africa, Asia, and Australia (Fig. 1; Table 1), reflecting the international nature of OA imaging research. With the exception of South America (and Antarctica), IWOAI has been to every continent, whereas other societies struggle to overcome their narrow one- or two-continent focus. As the meeting evolved, it developed a stronger publication record and online presence. Reports of the 2^nd^, 5^th^, 6^th^, 7^th^, and 18^th^ Workshops have been published [[1], [2], [3], [4], [5]]; the 6^th^ workshop being a joint event together with the 3^rd^ OA Biomarker Workshop. As innovative features, “Segmentation Challenges” were organized, one around the 13^th^ workshop in Charlottetown, Canada (2019) [5], and another one following later [6], and these were also published.

## The Website, ISOAI and the Journal of Osteoarthritis Imaging (OSTIMA)

The creation of the first IWOAI website in 2017 by Ali Guermazi and team was a true game changer for our small community. It provided a central platform, where information about previous and upcoming programs and workshop materials was shared. This online presence strengthened dissemination within the community, helped attract new researchers to the workshop, and provided a wonderful archive of memories for the participants.

As the workshop community expanded, the potential benefit of a more formal organizational structure became increasingly apparent. The process was not straightforward, as the bureaucratic and administrative hurdles are high. But Ali Guermazi took the baton where it was meant to go and spared no effort to make it happen. The process led to the establishment of the International Society for Osteoarthritis Imaging (ISOAI), based in Boston (MA, USA), in 2019. Under the guidance of the founding president, Ali Guermazi, ISOAI created an institutional framework that provided important continuity for the workshop series and for the OA imaging community. The formation of the society ensured continuity of the workshops as the field evolved. Further, the journal Osteoarthritis Imaging “OSTIMA” was initiated as an Open-Access-Journal and published its first issue in 2021, with Frank Roemer as Editor-in-Chief. The creation of the ISOAI and OSTIMA substantially strengthened the visibility of OA imaging science as a field, including broader recognition of imaging as a valid source of biomarkers alongside molecular and fluid-based markers, a term that for a long time was exclusively claimed by the molecular marker community [3]. The success and continuity of the workshop series owe much to this development, with OSTIMA publishing all conference abstracts labeled with an official digital object identifier (DOI) making these fully citable publications, and more recently a Special Issue of each meeting [5]. OSTIMA received approval to be indexed in PubMed Central (PMC), the most relevant indexing body for authors, in the spring of 2026, and thus all articles will now be listed in the PubMed database regardless of funding bodies. This is a result of a joint community effort and all authors who supported the journal from the beginning and believed in it success. This step has proven crucial in maintaining the momentum of the workshop series and ensuring its long-term sustainability.

## The 20th Workshop: Return to Bavaria

After the 19th meeting to have taken place in Cambridge (Fig. 1) IWOAI will celebrate its 20^th^ anniversary and reach an important milestone in 2026. Organized by Frank Roemer and Wolfgang Wirth, the meeting will take place near the English Garden in Munich, themed “Tissues: Linking Them All Together”. This reflects the long-standing recognition that OA affects the entire joint and that imaging may help to explore the interplay between tissues in OA.

Returning to Bavaria two decades after the first workshop provides a symbolic opportunity to reflect on how both the field and the workshop have evolved. As in previous years, the meeting will combine a strong scientific program with opportunities for informal interaction, including a full-day excursion to the famous Kloster Andechs and its “Biergarten”, continuing the traditions that have characterized IWOAI since its beginning.

## Outlook

Over the past two decades, OA imaging has evolved substantially. Quantitative MRI techniques have matured, automated image analysis methods have advanced rapidly, and imaging biomarkers have become important tools in OA clinical research.

Future progress will critically depend on integrating imaging with other sources of information, geno-, endo-, and phenotyping, in large population datasets, using new techniques such as Federated Learning. Advances in artificial intelligence and data science will further expand the possibilities for extracting important information from imaging data.

Continued interaction between academic, clinical and industry scientists, particularly learning to speak each other’s language, will remain crucial, as imaging biomarkers will play a central role in disease modifying OA drug (DMOAD) development, although two decades ago there was already optimism that effective structure-modifying therapies were within reach. Without DMOADs, there will be little imaging science in OA. This, of course, only partly depends on the measurement technology, but on the availability of effective agents.

Last, progress will depend on the regulatory environment which seems to be risk-averse and bureaucratic, and under these constraints drug development may become financially prohibitive, and even economically meaningless. Those who will suffer most will not be biotech or big pharma or the imaging science and biomarker research field, but it will be the patients suffering from OA who will have to continue living with this crippling disease, without hope of lasting improvement beyond pain management.

Finally, collegiality is of ever greater importance in an increasingly divided world, where far more can be achieved through collaboration than through division. The continued popularity and success of the IWOAI demonstrates the value of focused scientific meetings that foster interdisciplinary, international collaboration. As the workshop enters its third decade, it will hopefully continue to inspire new ideas, contribute to advances in OA research, and see the first structure-modifying therapy approved by regulators.

## Declaration of generative AI/AI-assisted technologies

During the preparation of this work, the author(s) used OpenAI (2026) ChatGPT Enterprise/Business based on GPT-5.4 [Large language model] https://chatgpt.com to search content, synthesize complex materials, gain an overview of the field, provide support in content organization, and improve language and readability. After using this tool, the author(s) thoroughly edited or rewrote the text and take full responsibility for the content of the published work.

## Consent for publication

All authors have consented to publication.

## Authors' contributions

• Study conception and design: FE, DH, AG
• Search and summary of material: FE
• Analysis & interpretation of data: FE, DH, AG
• Writing of the first manuscript draft: FE
• Critical manuscript revision and approval of final manuscript: FE, DH, AG

## Funding sources

Felix Eckstein receives direct funding from PROTO (EU Horizon), Berlin, Germany and indirect support from the Ludwig Boltzmann Institute of Arthritis and Rehabilitation (LBIAR), Vienna, Austria.

DH

AG work was supported by the NIH National Institute of Arthritis and Musculoskeletal and Skin Diseases (NIAMS) under Award Number R01AR079620-01

ROLE OF THE FUNDING SOURCES: The funding sources (PROTO and LBIAR) were not involved in the preparation or editing of this perspective manuscript.

EHTICS APPROVAL: NA.

## Editor Disclosure

The peer review process did not involve Editorial Board Member Ali Guermazi, and the editorial decision-making was led by editors not involved in the creation of this manuscript.

## Conflicts of interest

Felix Eckstein is CEO/CMO and co-owner of Chondrometrics GmbH; he has provided consulting services to Kolon-Tissuegene (KTG), Servier, Galapagos, Novartis, 4P Pharma, Formation Bio, Peptinov, Artialis, Sanofi, Argenx and Envilex outside the submitted work.

David Hunter provides consulting services and is member of advisory boards of: Haleon, Naia Health, Novartis, Lilly, Tissuegene, Doron, Sanofi, Formation Bio, and Enlivex

Ali Guermazi is President of BICL, LLC and Consultant to Novartis, TissueGene, Medipost, ICM, Peptinov, Scarcell, 4Moving, Paradigm, Pacira, Levicept, Coval, and Formation Bio.
